# Editorial: MISS innovations: Approaches, predictive outcomes, and risk avoidance

**DOI:** 10.3389/fsurg.2022.1105855

**Published:** 2023-01-09

**Authors:** Vadim A. Byvaltsev, Dino Samartzis, Morgan B. Giers

**Affiliations:** ^1^ Department of Neurosurgery, Irkutsk State Medical University, Irkutsk, Russia; ^2^ Department of Neurosurgery, Railway Clinical Hospital, Irkutsk, Russia; ^3^ Department of Traumatology, Orthopedic, and Neurosurgery, Irkutsk State Medical Academy of Postgraduate Education, Irkutsk, Russia; ^4^ Department of Orthopedic Surgery, Rush University Medical Center, Chicago, IL, United States; ^5^ School of Chemical, Biological, and Environmental Engineering, Oregon State University, Corvallis, OR, United States

**Keywords:** minimally invasive spine surgery, degenerative disc disease, spinal cord tumors, spine endoscopy, lumbar interbody fusion, spinal cord injury

**Editorial on the Research Topic** MISS innovations: Approaches, predictive outcomes, and risk avoidance

## Introduction

Since its inception, minimally invasive spine surgery (MISS) has divided the community of spinal surgeons into firm supporters and outspoken opponents. After several decades, the field has accumulated a significant body of research confirming the effectiveness of MISS techniques (1). However, MISS has long been subjected to considerable criticism and comprehensive analysis. Though criticism towards the minimally invasive field continues, we must admit that the techniques employed have stood the test of time and continue to conquer more areas of neurosurgery.

After analyzing various experiences with spinal interventions and data from the current literature, four criteria for compliance of the intervention with the MISS category were identified: anatomical, technical, human, and instrumental. Modern MISS has ample opportunities for medical imaging of the pathology and pre-operative planning of approach trajectory, which allows us to limit iatrogenic damage to the surrounding tissues. These interventions are realized with the help of modern specialized equipment: x-ray-transparent tables, electron-optical converters, neuron navigators, illumination and optical magnification systems, power equipment, robotic systems, complex retractor systems, and specialized instrumentation allowing work at any operative depth (2).

MISS has a long learning curve and requires diverse staffing and educational approaches from various fields including orthopedics, vascular surgery, and radiology. The need for these skills accounts for the increasing role of cadaver and simulation courses in minimally invasive techniques. The variety of integrated systems and their installation can add complexity to MISS. The changing technology requires additional education and training not only for the tools themselves, but also the most appropriate approach for a patient's unique anatomy while utilizing the tools. Additionally, the expanding selection of implants and materials, particularly bioactive materials, contribute to technique modification as well.

## Results

In this issue of Frontiers of Surgery: “MISS innovations: Approaches, Predictive Outcomes, and Risk Avoidance,” we are honored to present a collection of 14 publications that describe cutting-edge advances in MISS research and practice. These articles were selected through an open peer review process that brought together experts in spinal surgery, including 80 authors, 20 reviewers, and three editors.

In preparing this volume, the editorial team sought to highlight the ever-expanding field of applicational MISS techniques. Beginning as a separate set of techniques for the surgical treatment of degenerative diseases, MISS now tackles some of the most complex treatments, such as spinal oncology and severe spinal cord injury.

The first series of articles describes the results of minimally invasive techniques in the surgery of degenerative spinal lesions. The use of percutaneous transforaminal endoscopy under local anesthesia (3) demonstrates the technique's capabilities for patients with degenerative diseases of the lumbar spine to prevent nerve root damage and postoperative side effects.

In a cohort of patients with degenerative diseases of the cervical spine, Chen et al. (2022) studied the preliminary results of screwless cage placement. The satisfactory long-term clinical and radiological results can be summarized in future meta-studies (4).

Liang et al. (2022) investigated clinical outcomes and efficacy differences between paraspinal mini tubular lumbar decompression and minimally invasive transforaminal lumbar interbody fusion. The study considers the difference between the two concerning the treatment of degenerative grade I lumbar spondylolisthesis combined with spinal canal stenosis. The study shows that less extensive and costly treatment could be a viable primary surgical option for most patients.

Comparative studies are becoming increasingly important because of the variety of approaches to stabilizing intervention in patients with degenerative lumbar pathology. For example, Bokov et al. (2022) analyzed the potential effect of transforaminal lumbar interbody fusion (TLIF) vs. direct lateral interbody fusion (DLIF) on pedicle screw stability. The study demonstrated the advantage of DLIF technology in ensuring stability and reducing the frequency of revision interventions.

In contrast to the traditional transpedicular screw fixation technique, the cortical bone trajectory technique offers certain advantages. The reliability of screw placement along such a trajectory and prediction of screw loosening after single-level posterior lumbar interbody fusion using a nomogram was performed by Zhang et al. (2022).

While preparing this collection, we aimed to show an understanding of the significance and peculiarities throughout the lifetime of degenerative diseases of the spine in patients of older age. A systematic review, as seen in Techens et al. (2022) reflects the status of the increasingly popular lumbar cemented discoplasty technique. This technique can be used in elderly and high-risk polymorbid patients as a minimally invasive alternative to traditional spondylodesis.

In turn, a series of cases by Klimov et al. (2022) identified the main predictors of complications and adverse outcomes of minimally invasive surgical treatment in elderly patients with lumbar spine pathology.

The second block of articles is devoted to spinal neurooncology. Using minimally invasive approaches with tubular retractors can be used to optimize the surgical treatment of extramedullary tumors. For example, Kerimbayev et al. (2022) demonstrated the results of MISS for the treatment of dumbbell tumors with extra vertebral spread. The article shows a significant decrease in hospitalization time and postoperative pain syndrome compared to traditional open surgery. In addition, the long-term results of MISS techniques in the treatment of these types of tumors are studied by Pan et al. (2022).

To illustrate the possibilities of endoscopic techniques in spinal neurooncology surgery, the collection includes a case in Kravtsov et al. (2022) of successful percutaneous transforaminal endoscopic removal of neurinoma of the fifth lumbar spinal nerve using intraoperative neuromonitoring.

The neurooncology block concludes with a series by Yamada et al. (2022) emphasizing the importance of multimodal neurophysiological monitoring on the long-term outcomes of motor function after microsurgical resection for spinal cord tumors.

The block devoted to applying MISS technologies in patients with spinal cord injury presents a rare series, in Kravtsov et al. (2022) of three cases of lumbar and thoracic spinal bullet wounds sustained from firearms and traumatic weapons. Percutaneous endoscopic techniques were successfully used for bullet extraction from the spinal canal.

The collection concludes with two unique papers: a rare case of Crowned Dens Syndrome treatment with occipital-cervical fixation technology by Haas et al. (2022) and a literature review and the results of robotic technology in combination with MISS [Minimally Invasive Assisted Robotic Spine Surgery (MARSS)] seen in Pérez de la Torre et al. (2022).

## Summary

As MISS continues to face new challenges, the ever-expanding possibility of this technology requires further efficacy studies in new fields of spinal surgery. The current trend of modern spine surgery is the use of minimally invasive approaches, specialized retractors, and endoscopes combined with imaging and navigation systems (2). MISS will continue developing in new areas previously available only to traditional open surgery. We are proud to present our compendium reflecting these advances in spine surgery, delivered in the least invasive manner and accompanied by the best clinical outcomes and minimal surgical complications.
